# Oral Secondary Syphilis in an HIV-Positive Transgender Patient: A Case Report and Review of the Literature

**DOI:** 10.3390/dj11100231

**Published:** 2023-09-26

**Authors:** Rodolfo Mauceri, Martina Coppini, Antonio Cascio, Marcello Trizzino, Valentina Crivello, Ada Maria Florena, Giuseppina Campisi

**Affiliations:** 1 Department of Surgical, Oncological and Oral Sciences (DiChirOnS), University of Palermo, 90127 Palermo, Italy; rodolfo.mauceri@unipa.it (R.M.); giuseppina.campisi@policlinico.pa.it (G.C.); 2 Unit of Oral Medicine and Dentistry for Fragile Patients, Department of Rehabilitation, Fragility, and Continuity of Care, University Hospital Palermo, 90127 Palermo, Italy; 3 Department of Biomedical and Dental Sciences and Morphofunctional Imaging, University of Messina, 98122 Messina, Italy; 4 Department of Health Promotion Sciences, Maternal and Infant Care, Internal Medicine and Medical Specialties (ProMISE), University of Palermo, 90127 Palermo, Italy; antonio.cascio03@unipa.it (A.C.); marcello.trizzino@policlinico.pa.it (M.T.); valentina.crivello@unipa.it (V.C.); adamaria.florena@unipa.it (A.M.F.)

**Keywords:** syphilis, oral manifestation, oral ulcers, HIV, AIDS, sexually transmitted disease, transgender

## Abstract

Background: Syphilis is a worldwide sexually transmitted infection caused by Treponema pallidum. In most cases, the oral manifestations of syphilis infection are associated with cutaneous involvement. However, the present case report is noteworthy since the oral lesions are the sole clinical sign in an HIV-positive transgender patient. Case presentations: We reported an uncommon case of secondary syphilis in a 37-year-old seropositive transgender male, whose diagnostic suspect was based only on oral mucosal lesions. The patient was referred to the Oral Medicine Unit for the presence of multiple undiagnosed painful oral lesions. The intraoral examination revealed the presence of white and red plaques on the right and the left buccal mucosa and several painful lesions localized on the upper and lower labial mucosa. No cutaneous lesions were observed. Considering the sexual history of the patient and clinical findings, secondary syphilis infection was suspected. The serologic analysis was conducted, and the diagnosis of syphilis was confirmed. Moreover, to exclude the presence of oral epithelial dysplasia or malignant disease, an incisional biopsy was performed. Discussion: Compared to the literature data, oral lesions as lone signs of secondary syphilis infection are uncommon, especially in HIV-positive patients. Syphilis and HIV coinfection create a concerning situation as they interact synergistically, leading to an increased risk of transmission and faster disease progression. Conclusions: This case report emphasizes the importance of considering syphilis as a diagnostic possibility, even when oral lesions are the only clinical manifestations, especially in HIV-positive patients. Comprehensive evaluation, including a detailed sexual history and careful oral examination, is essential for accurate diagnosis and appropriate management in such cases.

## 1. Introduction

Syphilis is a worldwide sexually transmitted infection caused by Treponema pallidum. In the 15th century, syphilis was as a virulent and potentially fatal disease [1]. In 1943, the introduction of penicillin contributed to decreasing its incidence; however, in the past decade, syphilis infection remerged and is represented again as a public health issue [2].

The World Health Organization estimated approximately 7 million new syphilis infections worldwide in 2020 [3]. This resurgence can be attributed to several factors, including increased sexual promiscuity, unprotected oral sex, failure of condom use, the sex industry, and lack of relevant knowledge in developing countries [4].

Syphilis infection can be classified as either congenital or acquired. Acquired syphilis is divided into four stages: primary, secondary, latent, and tertiary. Each stage is characterized by different signs and symptoms [5,6]. Primary syphilis consists of the initial stage of infection and manifests itself with clear symptoms about three weeks after the inoculation [7]. Clinically, it is typically characterized by the presence of single or multiple circular and painless ulcers known as chancres, which commonly appear on the genital or anal regions. These chancres typically resolve spontaneously within two to eight weeks [8]. Usually, the diagnosis of primary syphilis is supported by laboratory investigations, which may include either positive serological tests or the direct examination of dark-field microscopy of scrapings obtained from the chancre [6]. In the absence of intervention, syphilis infection tends to advance to the secondary stage. Secondary syphilis is characterized by a wide variety of symptoms including mucocutaneous lesions, rash, low-grade fever, myalgia, and systemic lymphadenopathy [7]. In the presence of suspicion of secondary syphilis, the diagnosis is confirmed through a positive serological test [6]. In this stage, spirochetes can be found throughout the body whereafter spontaneous remission marks the start of the latent stage. Even with positive serology, latent syphilis consists frequently of an asymptomatic period of infection [9]. According to Canadian and United States guidelines, latent syphilis is divided into early latent when the infection was acquired within the past 12 months, and late latent if it has been present for longer [6]. Tertiary syphilis occurs usually decades after the initial infection, and it is primarily associated with cardiovascular and neurological complications. The hallmark of tertiary syphilis is the presence of gummatous lesions, which are necrotic granulomatous lesions pathognomonic of tertiary syphilis [7]. Untreated syphilis infection and its subsequent complications have been reported to be associated with a mortality rate ranging from 8% to 14% [10].

Given the wide variety of symptoms that characterize it, syphilis is also known as “the great imitator” [9]. The “great imitator” appears not only in the clinic but also in histopathology, since oral secondary syphilis may mimic various infectious, neoplastic, or immune-mediated processes [11]. Early diagnosis of syphilis infection is essential for adequate management, particularly in people living with HIV as it aids in appropriate management and prevents further complications.

Oral manifestations of syphilis can occur at any stage of the infection, although they commonly arise in secondary syphilis [10].

Secondary syphilis is a consequence of the hematogenous dissemination of the microorganisms, and it is characterized by systemic involvement. The patients may have symptoms such as fever, myalgia, headache, arthralgia, and generalized lymphadenopathy [5]. Secondary syphilis presents approximately 2–12 weeks after the primary stage with maculopapular skin rashes which may affect one or more areas of the body or mucous membrane lesions [4,12,13]. The most common cutaneous presentation is generalized, nonpruritic, symmetrical macular eruptions, which are frequently distributed on the trunk, palms, and soles, called syphilitic rosette [14].

In this case report, we present an uncommon case of secondary syphilis in an HIV-positive transgender patient where the suspicion diagnosis was initially solely based on the presence of oral lesions. Furthermore, we conducted a comprehensive literature review to provide a broader context for understanding this unusual presentation. This case highlights the importance of considering syphilis infection as a diagnostic possibility, even when oral lesions are the only apparent clinical signs, emphasizing the need for a high index of suspicion and thorough evaluation in at-risk populations.

## 2. Case Presentation

A 37-year-old HIV-positive transgender male was referred to the Oral Medicine Unit from the Infectious Diseases Unit (University Hospital “Paolo Giaccone” of Palermo, Palermo, Italy) for the presence of multiple painful oral lesions that had not been previously defined.

The patient declared that he was born in South America, in Colombia, and he was a cannabis smoker. The medical history of the patient revealed that he tested positive for HIV in April 2022 and subsequently initiated antiretroviral therapy with bictegravir/emtricitabine/tenofovir alafenamide (BIC/FTC/TAF) at doses of 50/200/245 mg.

The patient reported the appearance of recurrent oral lesions a few months earlier.

He also reported that these oral manifestations were usually associated with low-grade fever, general malaise, and feeding difficulty.

**Figure 1:**
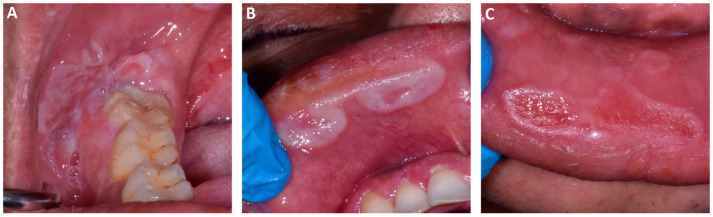
Clinical features at the first visit. The oral lesions were localized on the right buccal mucosa (**A**) and on the upper and lower labial mucosa (**B**,**C**).

During the extraoral examination, no lymphadenopathy and other clinical features were observed. The intraoral examination revealed a presence of white and red plaques on the right and the left buccal mucosa and several painful lesions localized on the upper and lower labial mucosa (Figure 1). No presence of cutaneous lesions on the body or other clinical signs were found or were reported.

Clinical features at the initial examination revealed oral lesions indicative of secondary syphilis. The oral lesions of secondary syphilis infection were localized on the right buccal mucosa (Figure 1A) and on the upper and lower labial mucosa (Figure 1B,C).

Based on the detailed sexual history of the patient, as well as the clinical findings, secondary syphilis infection was strongly suspected.

**Figure 2:**
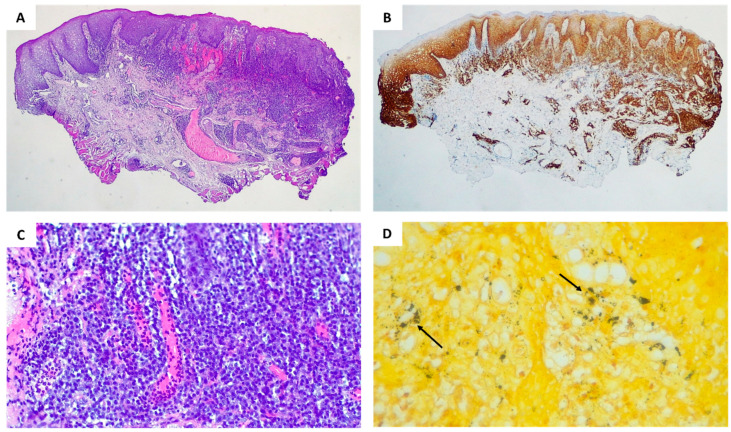
Histopathologic features of oral lesions localized on the lower labial mucosa revealed the superficial and deep perivascular along with perineural “lichenoid” inflammation associated to neoangiogenesis foci (H&E, original magnification 25×) (**A**); immunohistochemical study of CD138 (CD138 IHC stain, original magnification 25×) (**B**); high-power view of neoangiogenesis with marked endothelial swelling and neutrophilic vascular reaction (H&E, original magnification 200×) (**C**); Warthin Starry stain enhancement highlights the presence of dark agglomerates consisting of spirochetes (arrows) (Warthin Starry histochemical stain, original magnification 400×) (**D**).

The serologic analysis was performed, and the diagnosis of syphilis was confirmed. Additionally, to exclude the presence of oral epithelial dysplasia or malignant disease, an incisional biopsy from one oral lesion localized on the lower labial mucosa was conducted (Figure 2).

The histopathological examination revealed the presence of a “lichenoid” inflammation, consisting largely of plasma cells and, to a lesser extent, lymphocytes and histiocytes. Neoangiogenesis foci are visible in the superficial subepithelial connective tissue (Figure 2A). The immunohistochemical study of CD138 highlighted the prevalence of plasma cells in the inflammatory infiltrate and their perivascular and perineural disposition (Figure 2B). The high-power view showed neoangiogenesis with marked endothelial swelling and neutrophilic vascular reaction (Figure 2C). Worthy of note, the Warthin Starry stain enhanced the presence of dark agglomerates consisting of spirochetes (Figure 2D).

Additionally, the serologic tests for syphilis infection showed positivity for Treponema pallidum IgM and IgG antibodies (CLIA method). Additionally, the semi-quantitative non-treponemal test known as the Rapid Plasma Reagin (RPR) was positive with dilution 1:128.

The diagnosis of syphilis infection was confirmed. The patient received a single dose of 2.4 million units of benzathine penicillin G intramuscularly.

**Figure 3:**
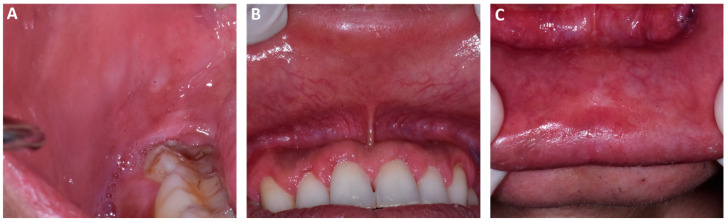
Clinical features of right buccal mucosa (**A**) and upper and lower labial mucosa (**B**,**C**) after 4 weeks of treatment.

One month later, the patient presented to our attention for a new oral examination. Encouragingly, the biopsy site had healed, and a significant improvement was observed in the previously afflicted oral lesions, as illustrated in Figure 3. Notable improvements were observed in the right buccal mucosa (Figure 3A), as well as in the condition of the upper and lower labial mucosa after 4 weeks of treatment (Figure 3B and Figure 3C, respectively).

The patient is still undergoing periodic follow-up visits.

## 3. Discussion

Syphilis is a sexually transmitted infection caused by Treponema pallidum [1].

Between 2000 and 2013, a noteworthy increase in diagnoses of syphilis infection has been observed occurring in developed countries, with a marked intensification in the proportion reported among Men who have Sex with Men (MSM) compared to non-MSM, rising from 26.8% to 55% [15]. This phenomenon may be the consequence of the decreasing AIDS-related mortality, attributed to the efficacy of HIV treatment in the past two decades [8]. This trend underscores the complex interplay between HIV and syphilis epidemiology, necessitating vigilant surveillance and targeted prevention efforts within this susceptible population.

Among risk factors for syphilis infection, HIV has been described in different studies [16,17]. HIV is a sexually transmitted infection, which affects the immune system by gradually destroying CD4 cells. Patients infected by HIV are more susceptible to developing many infections and some types of cancer [18]. Syphilis and HIV coinfection represent a condition to be allowed for since they interact synergically, increasing the risk of transmission, and accelerating the time of disease progression. Some studies suggest that HIV may alter the clinical presentation and natural history of syphilis infection increasing the risk of poor outcomes. These studies reported a higher probability of complications in these patients, including persistent chancres, rapid progression to gummatous disease, and a greater frequency of ocular involvement [19].

Understanding the intricate interplay between HIV and syphilis infections is crucial for optimizing clinical care and public health strategies. HIV-positive patients are more likely to develop neurosyphilis during the early stages of syphilis infection. Considering the dangerous synergy of syphilis and HIV, the UK Health Protection Agency has recommended annual serological testing for syphilis in all seropositive patients [16].

The clinical manifestations of syphilis are different according to the disease stage, including cutaneous lesions, musculoskeletal manifestations, ocular involvement, and neurological implications in the late stage [20]. Several studies have proposed a heightened susceptibility to neuro-ophthalmological complications in HIV-positive patients affected by syphilis infection [10,19].

Regarding the clinical findings of syphilis infection, oral manifestations are a well-documented occurrence in secondary syphilis, although they can manifest at any stage of the disease [5]. The clinical presentation of oral syphilis lesions is highly heterogeneous; for this reason, syphilis is often defined as “the great imitator” [9,11]. Generally, oral syphilis manifestations appear as erosive and/or ulcerative lesions or mucous patches [21]. The presented case is similar to other previous studies, since the patient showed white and pink lesions with a serpentine or spiral pattern [4,22,23].

The differential diagnosis of oral syphilis lesions includes drug-related ulcerations, psoriasis, tuberculosis, histoplasmosis, pemphigoid, lichen planus, and oral squamous cell carcinoma [24,25].

For this reason, while serological tests represent the gold standard for diagnosing syphilis, an oral biopsy was additionally conducted in this instance to exclude the potential presence of dysplasia or malignant disease in an HIV-positive patient.

Therefore, considering the significant variability in the clinicopathological presentation of oral syphilis, it is fundamental, in our opinion, to be aware of syphilis clinical and histopathological features in order to schedule all the necessary tests to achieve an early diagnosis and subsequent adequate management [11], for the reason that untreated syphilis infection can lead to irreversible neurological and cardiovascular complications, and it is associated with a reported mortality rate ranging from 8% to 14% [10].

Within the European community, rare are the reports of literature about oral manifestations of syphilis in HIV transgender patients, and no case, before the present one, was reported with the exclusive presence of oral signs of secondary syphilis infection [26,27].

The analysis of the literature on European patients highlighted only two Italian cases affected by syphilis infection with the presence of oral lesions [12,28].

Compilato et al. described a case of a 45-year-old man affected by syphilis whose diagnosis was based on an unusual lesion on the oral mucosa associated with ulcerative lesions on the glans and a symmetrical maculopapular rash affecting the palms, soles, and trunk of the body [28].

Furthermore, Leuci et al. conducted a study involving 12 patients with oral manifestations of syphilis infection. However, only three of the enrolled patients presented oral lesions as the unique sign of disease [12]. It is noteworthy that none of the cases featured seropositive or transgender patients.

Like in the present case, in some studies, patients suffering from oral lesions as the only sign of secondary syphilis infection had been reported, but among these there were no HIV-positive patients [22,29].

In a multicenter study conducted by De Andrade et al., cutaneous manifestations were observed only in 33.7% of patients affected by secondary syphilis [30].

Moreover, in a study conducted in Southern Brazil on 105 patients affected by syphilis diagnosed from oral manifestations, only 39.4% of them showed or reported cutaneous signs [21].

Therefore, even if oral manifestations of syphilis are commonly associated with mucocutaneous and maculopapular lesions, it is important to take into consideration that in some cases, oral ulcerations may be the exclusive manifestations of infection [4].

To our knowledge, to date in the literature, only one study has described the manifestations of oral syphilis infection in patients living with HIV. However, it is important to note that many of the patients reported in that study exhibited additional cutaneous signs, such as a maculopapular rash on the trunk, palmar and plantar lesions, and alopecia in the eyebrows and eyelashes, in addition to the oral lesions [11]. In this recent observational study, the authors highlighted a predilection of oral syphilis lesions for specific oral sites, in detail, the soft palate, uvula, and oropharynx, followed by the tongue, hard palate, buccal mucosa, and labial mucosa. Additionally, in contrast to the presented symptomatic case, 95.7% of the cases reported by Ramírez-Amador et al. were asymptomatic [11].

These uncommon cases underscore the necessity for healthcare providers to maintain a high degree of suspicion, even when oral lesions appear isolated, in individuals with potential syphilis exposure, emphasizing the significance of a detailed medical and sexual history of the patient and a meticulous oral examination.

Due to the limitations related to the presentation of a single case, a review of the literature has been performed in order to highlight the characteristics of patients affected by syphilis oral lesions, comparing the results obtained to the features of our patient. Another limitation may be related to the sexual identification of our patient, however, even if our patient may represent a small percentage of the population, the uniqueness of his case had us oriented to some suspicious diagnoses, including syphilis.

In light of the literature evidence of increasing cases of syphilis infection, it is imperative to expand efforts in syphilis prevention. This could include rigorous screening programs targeting high-risk populations to improve early diagnosis and intervention during the early stages of syphilis infection. Early identification and treatment can significantly mitigate disease progression and the occurrence of complications, as well as the incidence of syphilis infection. Furthermore, it is essential to invest in research funding to develop an effective syphilis vaccine, which could ultimately offer a powerful tool for syphilis prevention and treatment on a larger scale.

## 4. Conclusions

The present case showed very uncommon concurrent conditions, indeed, the presence of oral lesions in the absence of simultaneous cutaneous involvement was the only sign of syphilis in an HIV-positive patient.

A detailed medical history is the first and fundamental step in every diagnostic process. In the present case, a detailed medical and sexual history of patients raised the suspicion of syphilis, even if in the presence of exclusive oral lesions.

Due to the potentially fatal event related to syphilis progression, clinicians should be aware of the oral manifestations of syphilis, considering that a comprehensive anamnesis and a meticulous oral examination could play a crucial role in its early diagnosis.

## Data Availability

All data generated or analyzed during this study are included in this published article.
